# Left atrial size and risk of recurrent ischemic stroke in cardiogenic cerebral embolism

**DOI:** 10.1002/brb3.1798

**Published:** 2020-08-12

**Authors:** Weiwei Quan, Xuezhi Yang, Youyu Li, Jia Li, Weiyi Ye, Ou Zhang, Xu Zhang

**Affiliations:** ^1^ Department of Neurology the First Affiliated Hospital of Wenzhou Medical University Wenzhou China; ^2^ Department of Emergency Medicine the Second Affiliated Hospital and Yuying Children’s Hospital of Wenzhou Medical University Wenzhou China

**Keywords:** cardiogenic cerebral embolism, left atrial size, recurrent ischemic stroke, transthoracic echocardiography, Weiwei Quan is first author

## Abstract

**Background:**

Left atrial enlargement (LAE) was reported to be associated with ischemic stroke and its recurrence. Limited data are available on the relationship of LAE and cardiogenic cerebral embolism (CCE). Our aim is to access the association of left atrial size and the recurrence of ischemic stroke in CCE.

**Methods:**

We prospectively included 303 CCE patients who underwent transthoracic echocardiography (TTE). Left atrial size was estimated with left atrial diameter (LAD), diameter/height (LAD/H), and left atrial diameter/body surface area (LAD/BSA). The endpoint was one‐year recurrent ischemic stroke. Cox proportional hazard models were performed to access the association between left atrial size and recurrent ischemic stroke.

**Results:**

During follow‐up, 27 patients suffered recurrent ischemic stroke. In multivariate COX regression models adjusted for confounders including age, gender, hypertension, diabetes, and history of stroke or transient ischemic attack (TIA), platelet count, fasting blood glucose (FBG), antithrombotic drugs at discharge, stroke volume, and cardiac output, LAD, LAD/H, and LAD/BSA all were independent risk factors of recurrent ischemic stroke [LAD: HR 1.065, 95% CI (1.006–1.128), *p* = .029; LAD/H: HR 1.157, 95% CI (1.066–1.255), *p* < .001; LAD/BSA: HR 1.128, 95% CI (1.059–1.202), *p* < .001]. Receiver‐operator characteristic curves showed that LAD/BSA had better predicting effect. The area under the curve (AUC) was 0.543 [95%CI (0.444–0.642), *p* = .461) for LAD, 0.626 [95%CI (0.530–0.723), *p* = .03] for LAD/H, and 0.655 [95%CI (0.558–0.752), *p* = .008] for LAD/BSA.

**Conclusion:**

LAE is an independent risk factor for one‐year recurrence of ischemic stroke in patients with CCE.

## Graphical abstract

Cardiac ultrasound parameters and other clinical data of patients with cardiogenic cerebral embolism were collected. The endpoint was one‐year recurrent ischemic stroke. We found that left atrial enlargement is an independent risk factor for one‐year recurrence of ischemic stroke in patients with cardiogenic cerebral embolism.

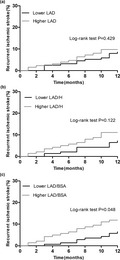

## 1 INTRODUCTION

Stroke is the second leading cause of death and the third most common cause of disability worldwide (Feigin, Norrving, & Mensah, 2017). Especially in China, it has surpassed ischemic heart disease as the first leading cause of death (Yang et al., 2013). Ischemic stroke was divided into five categories according to the Trial of Org 10,172 in Acute Stroke Treatment (TOAST) criteria: large artery atherosclerosis (LAA), cardioembolism, small‐vessel occlusion, stroke of other determined etiology, and stroke of undetermined etiology (Adams et al., 1993). Cardiogenic cerebral embolism (CCE) is the most severe subtype and accounts for about 14%–30% of all ischemic stroke types, which is characterized by severe clinical symptom, high mortality, and high rate of relapse (Arboix & Alio, 2010).

The left atrium is a predilection site for cardiac thrombosis. Left atrial enlargement (LAE) is suggested to be closely associated with left atrial thrombosis in research of patients with atrial fibrillation (AF) (Ayirala, Kumar, O'Sullivan, & Silverman, 2011; Malik et al., 2015). Hemodynamic disorders and other factors such as myocardial remodeling, inflammatory response, and activation of coagulation are important mechanism in pathogenesis of thrombosis (Ayirala et al., 2011; Warraich, Gandhavadi, & Manning, 2014). Many studies have shown a close correlation between LAE and ischemic stroke (Barnes et al., 2004; Bouzas‐Mosquera et al., 2011; Nagarajarao et al., 2008; Shaikh et al., 2013). It was found that this correlation was different in different types of stroke, with mild left atrial expansion associated with the occurrence of atherosclerotic stroke, while further expansion increased the risk of cardioembolic stroke (Shaikh et al., 2013). Recently, several studies showed that LAE was associated with recurrent ischemic stroke (Paciaroni et al., 2016; Xue et al., 2017; Yaghi et al., 2015). Yaghi et al. suggested that LAE was only associated with the risk of recurrent cardioembolic and cryptogenic stroke, but not all types of recurrent ischemic stroke (Yaghi et al., 2015). Another prospective study found that LAE increased the risk of recurrent ischemic stroke, and the correlation was more prominent in patients with cardioembolic and cryptogenic stroke (Xue et al., 2017). We expect that the left atrium size may be a valuable predictor of recurrent ischemic stroke in patients with CCE. The aim of this study was to investigate the association between left atrial size and the recurrence of ischemic stroke in patients with CCE.

## 2 METHODS

### 2.1 Study population

Between November 2015 and October 2016, 339 consecutive CCE patients admitted to the First Affiliated Hospital of Wenzhou Medical University were entered in our prospective collected database. The diagnostic criteria for CCE referred to TOAST system (Adams et al., 1993): patients with CCE presumably due to an embolus arising in the heart. (1) At least one correlated cardiac source for an embolus must be identified; (2) Clinical and imaging manifestations are similar to LAA ischemic stroke; (3) Evidence of a previous transient ischemic attack (TIA) or stroke in > 1 vascular territory or systemic embolism supports a clinical diagnosis of cardiogenic stroke.

We further excluded 7 patients who had malignant tumor, 30 patients for lack of important medical information such as cardiac ultrasound parameters and brain imaging examination, 1 patient who were admitted to our hospital after 30 days of onset, 1 patient who had severe liver and kidney dysfunction. Thus, 303 people were finally included in the analysis. This study was approved by the ethics committee of the First Affiliated Hospital of Wenzhou Medical University. All patients or their relatives signed the informed consent in this study.

### 2.2 Clinical data

Demographic data, past medical history, and clinical information including National Institutes of Health Stroke Scale (NIHSS) score, laboratory data, imaging data, ultrasound parameters, and discharge medication were collected. Patients were divided into nonsmokers, former smokers, and current smokers (Bejot et al., 2014). Nonsmokers were defined as those who had never smoked, former smokers were defined as those who had not smoked for more than three months, and current smokers were defined as those who kept smoking. Hypertension was defined as systolic blood pressure (BP) ≥ 140 mmHg or diastolic BP ≥ 90 mmHg, or a previously established hypertensive diagnosis. Diabetes mellitus was defined as fasting blood glucose (FBG) level ≥ 126 mg/dl (7 mmol/L), nonfasting glucose level ≥ 200 mg/dl (11.1 mmol/L), or a previously established diabetic diagnosis. Classification of cerebral infarction size (Paciaroni et al., 2015): (1) small, the maximum diameter of lesion was ≤ 1.5 cm; (2) medium, a lesion was in a cortical superficial branch of the anterior cerebral artery (ACA) or posterior cerebral artery (PCA), in one cortical superficial branch of middle cerebral artery (MCA), in the deep branch of MCA, in the internal border zone territories; (3) large, a lesion involved the complete territory of ACA, MCA, or PCA, in two cortical superficial branches of MCA, in a cortical superficial branch of MCA which was associated to the MCA deep branch, in more than one large artery territory, or a lesion's dimeter ≥ 1.5 cm in the brain stem or cerebellum.

### 2.3 Echocardiography measurements and parameters

Transthoracic echocardiography (TTE) was performed in all subjects with a GE Vivid 7 Dimension system, which was performed in the left lateral decubitus position using standard imaging planes, according to the recommendations of the American Society of Echocardiography (Sahn, DeMaria, Kisslo, & Weyman, 1978). The size of the left atrium is closely related to the body size of the individual (Pritchett et al., 2003). The diameter was then normalized by the subject's height (Gerdts et al., 2007; Nagarajarao et al., 2008; Xue et al., 2017) and body surface area (BSA) (Pritchett et al., 2003; Xue et al., 2017). Left ventricular ejection fraction (LVEF) was estimated with the Teichholz formula or the Simpson rule. Ventricular septal thickness, left ventricular (LV) posterior wall thickness, stroke volume, and cardiac output were also measured.

### 2.4 Follow‐up

Taking the onset time as the starting point, the patients were followed up regularly (3 months, 6 months, and 1 year). The time error was no more than 10 days. They were followed up through face‐to‐face or telephone conversations. The endpoint of the study was recurrent ischemic stroke. A total of 36 patients lost to follow‐up were censored. Among them, 18 patients were lost due to telephone loss and refusal to visit, and 28 patients were lost due to death from a nonendpoint event.

### 2.5 Statistical analysis

Categorical variables were presented as number (percentage) and analyzed by chi‐square or Fisher's exact test. Distribution of continuous variables was tested by Kolmogorov–Smirnov test. Continuous variables accorded with normal distribution were presented as mean ± standard deviation (*SD*) and analyzed by Independent‐samples *t* test, while those not complied with normal distribution were presented as medians (interquartile range, IQR) and analyzed by Mann–Whitney *U* test. Parameters including LAD, left atrial diameter/height (LAD/H), left atrial diameter/body surface area (LAD/BSA) were analyzed as independent variables in analyses, respectively. Cox proportional hazard models were fitted to access the association between left atrial size and recurrent ischemic stroke, unadjusted, and adjusted for demographic characteristics (age, gender) and related risk factors (age, gender, hypertension, diabetes, history of stroke, or TIA, platelet count, FBG, antithrombotic drugs at discharge, stroke volume, and cardiac output). Hazard ratio (HR) and 95% confidence interval (95% CI) were estimated. Cumulative event curves were generated with the Kaplan–Meier method and compared by the log‐rank test. The predictive value of left atrial size for recurrent ischemic stroke was analyzed by receiver operating characteristic (ROC) curve analysis. Statistical analyses were performed using Statistical Program for Social Sciences (SPSS) software (version 19.0, SPSS Inc). Cumulative event curves were created by GraphPad Prism 5 software. *p* < .05 was considered statistically significant.

## 3 RESULTS

### 3.1 Clinical characteristics

The demographic and clinical characteristics of all the study subjects were summarized in Table 1. A total of 303 patients were enrolled in the analysis, including 180 males (59.4%) and 123 females (40.6%). The mean age of the patients was 72.29 ± 10.33 years, and 16.2% of them had experienced ischemic stroke or TIA before. The median NIHSS score was 8 (3, 15). During one year of follow‐up, end point event of a recurrent ischemic stroke happened in 27 patients. Table 2 showed the baseline characteristics and echocardiographic parameters of patients with and without recurrent ischemic stroke. A higher LAD level was shown in the recurrent group in the comparison of these two groups, but there was no statistical difference between the two groups [47 mm, IQR (43, 51) versus. 45 mm, IQR (42, 51), *p* = .461]. Similarly, LAD/H was higher in patients with recurrent ischemic stroke that was not statistically significant [29.57 ± 3.15 mm/m versus. group: 28.04 ± 4.48 mm/m, *p* = .084]. But, LAD/BSA was found observably higher in the patients with recurrent ischemic stroke than patients without recurrent ischemic stroke and it remained statistically significant [29.14 mm/m^2^, IQR (26.18, 31.87) versus. 26.75 mm/m^2^, IQR (23.96, 29.82), *p* = .008]. In addition, the recurrent group had lower stroke volume level than the nonrecurrent group [60.10 ml, IQR (53.50,65.40) versus. 68.25 ml, IQR (56.15,80.53), *p* = .015], and antithrombotic drugs at discharge of the two groups were significantly different (*p* = .021). Comparative results of other baseline indicators were also presented in Table 2.

**Table 1:** Demographics and clinical characteristics of study patients

	Total (*n* = 303)
Age, year, mean (*SD*)	72.29 ± 10.33
Gender (male), *n* (%)	180 (59.4)
Smoking status, *n* (%)	
Nonsmokers	213 (70.3)
Former smokers	39 (12.9)
Current smokers	51 (16.8)
History of drinking, *n* (%)	92 (30.4)
Hypertension, *n* (%)	244 (80.5)
Diabetes, *n* (%)	91 (30.0)
History of stroke or TIA, *n* (%)	49 (16.2)
NIHSS, median (IQR)	8 (3.15)
Infarction size, *n* (%)	
Small	39 (12.9)
Medium	122 (40.3)
Large	142 (46.9)
Hemorrhagic transformation, *n* (%)	112 (36.96)
Intravenous thrombolysis or arterial intervention, *n* (%)	56 (18.5)
Antithrombotic drugs at discharge, *n* (%)	
None	55 (18.2)
Antiplatelet	85 (28.1)
Anticoagulant	163 (53.8)

Abbreviations: *SD*, standard deviation; IQR, inter quartile range; TIA, transient ischemic attack; NIHSS, National Institutes of Health Stroke Scale.

**Table 2:** Characteristics and echocardiographic parameters of patients with and without recurrent ischemic stroke

Characteristics	Recurrent ischemic stroke (*n* = 27)	Nonrecurrent ischemic stroke (*n* = 276)	*p*
Age, year, mean (*SD*)	74.63 ± 8.02	72.07 ± 10.51	.219
Gender (male), *n* (%)	17 (63.0)	163 (59.1)	.693
Smoking status, *n* (%)			
Nonsmokers	16 (59.3)	197 (71.4)	.358
Former smokers	4 (14.8)	35 (12.7)	
Current smokers	7 (25.9)	44 (15.9)	
History of drinking, *n* (%)	7 (25.9)	85 (30.8)	.599
Hypertension, *n* (%)	20 (74.1)	224 (81.2)	.375
Diabetes, *n* (%)	11 (40.7)	80 (29.0)	.203
History of stroke or TIA, *n* (%)	7 (25.9)	42 (15.2)	.169
Systolic BP, mmHg, mean (*SD*)	149.56 ± 23.61	146.22 ± 21.73	.450
Diastolic BP, mmHg, mean (*SD*)	81.78 ± 12.82	83.28 ± 14.84	.611
NIHSS, median (IQR)	6 (3,11)	9 (3, 15.75)	.380
WBC, ×109/L, median (IQR)	7.10 (6.47, 10.14)	7.60 (6.23, 9.66)	.926
Platelet count, ×109/L, mean (*SD*)	173.15 ± 38.43	189.13 ± 58.20	.058
Fibrinogen, g/L, median (IQR)	3.53 (2.78, 4.37)	3.48 (2.95, 4.13)	.942
FBG, mmol/L, median (IQR)	6.00 (4.90, 8.00)	5.25 (4.60, 6.40)	.088
TG, mmol/L, mean (*SD*)	4.27 ± 1.18	4.62 ± 1.22	.156
TC, mmol/L, median (IQR)	1.05 (0.93, 1.39)	1.25 (0.93, 1.56)	.289
HDL, mmol/L, mean (*SD*)	1.04 ± 0.25	1.14 ± 0.31	.102
LDL, mmol/L, mean (*SD*)	2.61 ± 1.04	2.73 ± 0.95	.529
Infarction size, *n* (%)			
Small	3 (11.1)	36 (13.0)	.888
Medium	12 (44.4)	110 (39.9)	
Large	12 (44.4)	130 (47.1)	
Hemorrhagic transformation, *n* (%)	11 (40.7)	101 (36.6)	.670
Intravenous thrombolysis or arterial intervention, *n* (%)	6 (22.2)	50 (18.1)	.605
Antithrombotic drugs at discharge, *n* (%)			
None	6 (22.2)	49 (17.8)	.021
Antiplatelet	13 (48.1)	72 (26.1)	
Anticoagulant	8 (29.6)	155 (56.2)	
Echocardiographic parameters			
LAD，mm, median (IQR)	47 (43, 51)	45 (42, 51)	.461
LAD/H, mm/m, mean (*SD*)	29.57 ± 3.15	28.04 ± 4.48	.084
LAD/BSA, mm/m^2^, median (IQR)	29.14 (26.18, 31.87)	26.75 (23.96, 29.82)	.008
Ventricular septal thickness, mm, median (IQR)	10 (10, 11)	11 (10, 12)	.472
LV posterior wall thickness, mm, median (IQR)	10 (10, 11)	10 (10, 11)	.681
Stroke volume, ml, median (IQR)	60.10 (53.50, 65.40)	68.25 (56.15, 80.53)	.015
Cardiac output, L/min, median (IQR)	4.80 (3.90, 5.80)	5.30 (4.20, 6.50)	.051
LVEF, %, median (IQR)	62.10 (54.20, 66.90)	62.30 (56.33, 67.40)	.736

Abbreviations: *SD*, standard deviation; IQR, inter quartile range; TIA, transient ischemic attack; BP, blood pressure; NIHSS, National Institutes of Health Stroke Scale; WBC, white blood cell; FBG, fasting blood glucose; TG, total cholesterol; TC, triglyceride; HDL, high‐density lipoprotein; LDL, low‐density lipoprotein; LAD, left atrial diameter; LAD/H, left atrial diameter/height; LAD/BSA, left atrial diameter/ body surface area; LV, left ventricular; LVEF, left ventricular ejection fraction.

### 3.2 **Association between left atrial size and one‐year recurrent ischemic strok**e

**Figure 1:**
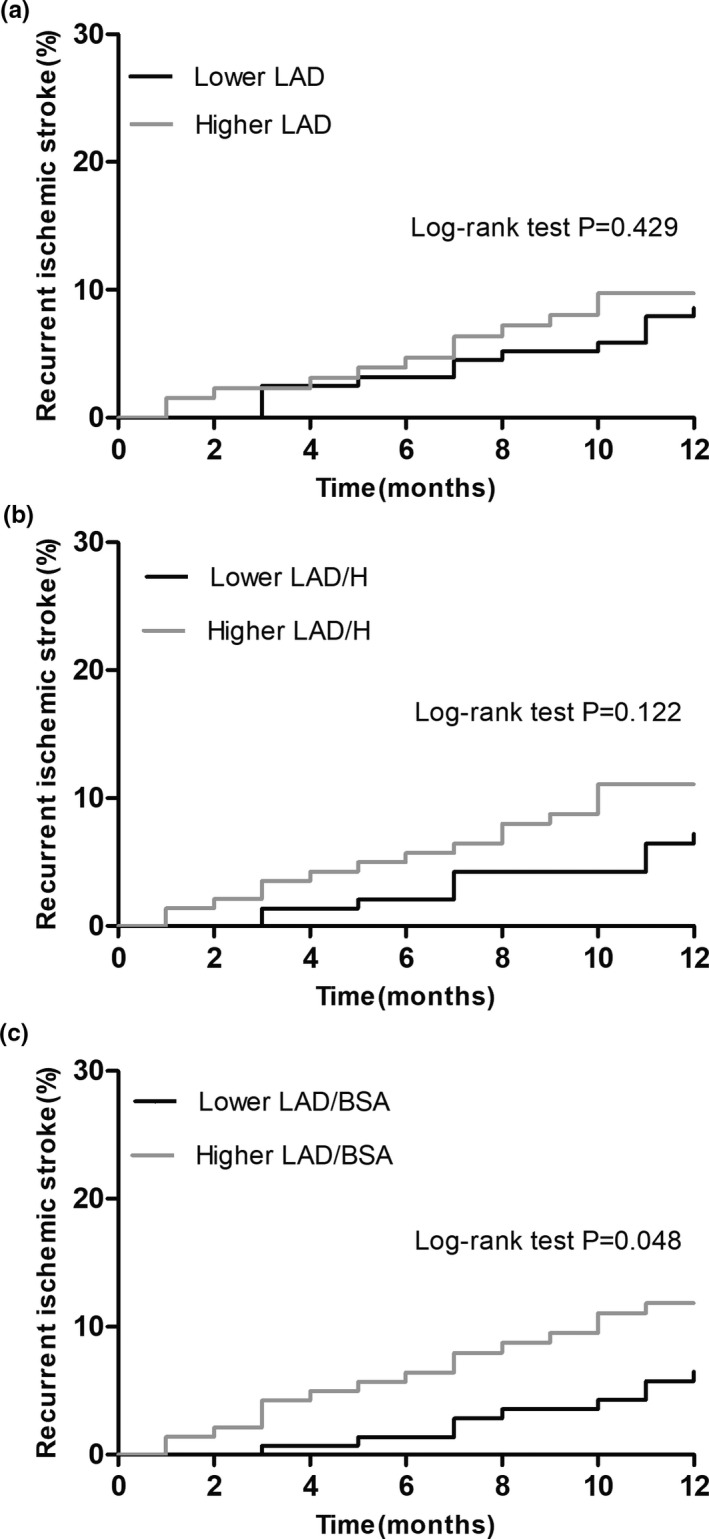
Cumulative recurrence probability of ischemic stroke according to left atrium size. LAD, left atrial diameter; LAD/H, left atrial diameter/height; LAD/BSA, left atrial diameter/ body surface area

The results of univariate and multivariate analyses based on Cox proportional hazards models were shown in Table 3. In an unadjusted model, LAD and LAD/H were not significantly associated with the risk of recurrent ischemic stroke [LAD: unadjusted HR 1.014, 95% CI (0.962–1.069), *p* = .612; LAD/H: unadjusted HR 1.070, 95% CI (0.995–1.150), *p* = .068], while LAD/BAS level was significantly associated with the risk of recurrent ischemic stroke [unadjusted HR 1.070, 95% CI (1.009–1.135), *p* = .024]. Demographic characteristics including age and gender were adjusted in the Model 2, in which LAD was not significantly associated with the risk of recurrent ischemic stroke [adjusted HR 1.015, 95% CI (0.961–1.072), *p* = .587]. But, higher levels of LAD/H and LAD/BSA were associated with higher risk of recurrent ischemic stroke after adjusting for age and gender [adjusted HR1.158, 95% CI (1.002–1.170), *p* = .045; adjusted HR 1.084, 95% CI (1.017–1.155), *p* = .013]. In the Model 3, risk factors (age, gender, hypertension, diabetes, and history of stroke or TIA) and baseline factors with a *p*‐value < .1 in univariate analyses (platelet count, FBG, antithrombotic drugs at discharge, stroke volume, and cardiac output) were adjusted in the multivariate COX regression analyses. It was shown that LAD, LAD/H, and LAD/BSA as continuous variables all were associated with the risk of recurrent ischemic stroke [LAD: adjusted HR 1.065, 95% CI (1.006–1.128), *p* = .029; LAD/H: adjusted HR 1.157, 95% CI (1.066–1.255), *p* < .001; LAD/BSA: adjusted HR 1.128, 95% CI (1.059–1.202), *p* < .001]. Cumulative recurrence probability of ischemic stroke according to left atrium size was presented in Figure 1. The left atrial size parameters were divided into two groups by the median, respectively (LAD: median 46, LAD/H: median 27.88, LAD/BSA: median 26.93). Kaplan–Meier curves showed significantly higher recurrence probability of ischemic stroke in higher LAD/BSA group (*p* < .05, Figure 1c).

**Table 3:** Association of left atrial size and recurrent ischemic stroke

Left atrial size	Model 1, Unadjusted		Model 2^a^		Model 3^b^	
	HR (95% CI)	*p*	HR (95% CI)	*p*	HR (95% CI)	*p*
LAD	1.014 (0.962–1.069)	.612	1.015 (0.961–1.072)	.587	1.065 (1.006–1.128)	.029
LAD/H	1.070 (0.995–1.150)	.068	1.158 (1.002–1.170)	.045	1.157 (1.066–1.255)	<.001
LAD/BSA	1.070 (1.009–1.135)	.024	1.084 (1.017–1.155)	.013	1.128 (1.059–1.202)	<.001

Abbreviations: LAD, left atrial diameter; LAD/H, left atrial diameter/height; LAD/BSA, left atrial diameter/ body surface area.

^a^ Adjusted for age and gender.

^b^ Adjusted for age, gender, hypertension, diabetes, history of stroke, or transient ischemic attack, platelet count, fasting blood glucose, antithrombotic drugs at discharge, stroke volume, and cardiac output.

### 3.3 ROC analysis

**Figure 2:**
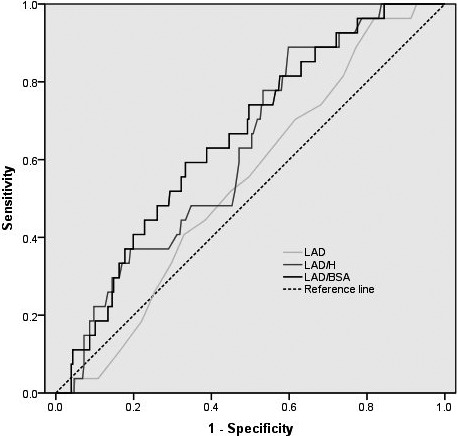
Receiver‐Operator Characteristic Curves for left atrial size to predict Recurrent ischemic stroke. LAD, left atrial diameter; LAD/H, left atrial diameter/height; LAD/BSA, left atrial diameter/ body surface area

ROC curves were displayed in Figure 2. Comparison of the LAD, LAD/H, and LAD/BSA showed that LAD/BSA was the best predictor of one‐year recurrent ischemic stroke in CCE patients. The area under the curve (AUC) was 0.543 [95%CI (0.444–0.642), *p* = .461) for LAD, 0.626 [95%CI (0.530–0.723), *p* = .03] for LAD/H, and 0.655 [95%CI (0.558–0.752), *p* = .008] for LAD/BSA. The cutoff value of LAD/BSA was 28.38 mm/m^2^ with a sensitivity of 59.3% and specificity of 66.7%.

## 4 DISCUSSION

This study shows that LAE is a significant risk factor for recurrence of ischemic stroke in patients with CCE, and the parameter of LAD/BSA has better predictive value than LAD/H and LAD. Since the left atrium size is closely related to the body size of individuals (Pritchett et al., 2003), the left atrial size was estimated by indicators including LAD, LAD/H (Gerdts et al., 2007; Nagarajarao et al., 2008; Xue et al., 2017), and LAD/BSA (Pritchett et al., 2003; Xue et al., 2017). Volume and diameter are common indicators reflecting the size of the left atrium. Some studies showed that compared with the LAD, the left atrial volume was more accurate in reflecting the size of the left atrium and more closely related to cardiovascular events (Pritchett et al., 2003; Tsang et al., 2006). But LAD is more clinically available, and also considered as a good indicator of left atrium size, which is closely related to cardiovascular and cerebrovascular diseases (Bouzas‐Mosquera et al., 2011; Kizer et al., 2006; Yaghi et al., 2015).

Many population‐based studies have reported the association between left atrial size and ischemic stroke. A study based on a population of nonatrial fibrillation showed that left atrial volume was an independent predictor for first ischemic stroke after adjusting for other potential risk factors (Barnes et al., 2004). It was also suggested that left atrial volume indices were linked to specific stroke phenotype (Shaikh et al., 2013). Alberto et al. found that LAD had a graded and independent association with ischemic stroke in women (Bouzas‐Mosquera et al., 2011). In addition, the Atherosclerosis Risk in Communities (ARIC) study (Nagarajarao et al., 2008) showed that left atrial size, estimated by LAD/H, was a significant predictor of both ischemic stroke and all‐cause death after adjusting for traditional cardiovascular risk factors. However, after further adjustment of LV hypertrophy and low LVEF, the correlation between LAD/H and ischemic stroke was significantly weakened. Oppositely, in a study exploring the association between cardiac ultrasound variables and cardiovascular events, no correlation between LAD and stroke was found (Gardin et al., 2001).

The association between LAE and recurrent ischemic stroke has also been proved in several studies (Paciaroni et al., 2016; Xue et al., 2017; Yaghi et al., 2015). In a study based on patients with AF‐associated acute ischemic stroke, LAE, especially severe, was shown to be an independent marker for recurrent ischemic stroke and systemic embolism (Paciaroni et al., 2016). The Northern Manhattan Stroke study showed that moderate to severe LAE evaluated by LAD was associated with the risk of recurrent cardioembolic and cryptogenic stroke (Yaghi et al., 2015). Similarly, another study indicated that LAE and recurrence of ischemic stroke were more closely related in patients with cardioembolic and cryptogenic stroke than other types, in which the left atrial size was estimated by the two indicators LAD/H and LAD/BSA (Xue et al., 2017).

The mechanisms accounting for the association of LAE with recurrence of ischemic stroke have not been fully clarified. The enlargement of left atrial leads to changes in hemodynamics and decreased blood flow velocity in the left atrial appendage, which may increase the risk of thrombosis and embolism (Yaghi et al., 2015). It was suggested that increased preload of left atrial lead to atrial myocardial remodeling such as intimal injury, atrial fibrosis, and destruction of muscle bundle structure, which might further activate platelet adhesion aggregation (Tab ata et al., 1996). In addition, the correlation between LAE and AF has long been reported, and there should be an interaction between the two (Dittrich et al., 1999; Parkash et al., 2004; Vaziri, Larson, Benjamin, & Levy, 1994), which may in turn increase the risk of ischemic stroke. The Framingham study showed that LAE was an independent risk factor for AF. For every 5 mm increase in LAD, the HR of AF was 1.39 (Vaziri et al., 1994). Research showed that LAE would increase the incidence of left atrial appendage thrombosis in patients with AF (Ayirala et al., 2011; Malik et al., 2015). What's more, pathological factors that affect left atrial pressure load and volume load would cause expansion of left atrium, and the size of left atrial may reflect the severity of cardiovascular diseases such as mitral valve disease, cardiomyopathy, coronary heart disease, and hypertension to some extent (Douglas, 2003; Messika‐Zeitoun et al., 2007). Therefore, LAE might be an indirectly risk factor for recurrence of ischemic stroke.

There are some limitations existing in this study. First, LAD and LAD indexes were used as indicators to assess the size of left atrial in this study, while the left atrial volume, which was considered to be a more accurate indicator for left atrial size, was not measured in this study. Second, the sample size of this study is small, which may reduce the reliability of statistical results. Third, since most of the follow‐up results were obtained in outpatient or telephone, it is difficult to accurately assess the specific type of recurrent ischemic stroke.

## 5 CONCLUSION

In conclusion, this is the first study to analyze the correlation between left atrial size and recurrence of ischemic stroke based on CCE population. We found that LAE is an independent risk factor for one‐year recurrence of ischemic stroke in patients with CCE. Further research based on large samples is warranted to explore the prognostic utility of left atrial size in patients with CCE and to clear whether other indicators of left atrial size such as left atrial volume can improve the prediction effect.

## CONFLICT OF INTEREST

The authors declare that they have no conflict of interest.

## AUTHOR CONTRIBUTIONS

Weiwei Quan and Xu Zhang designed the study. Weiwei Quan, Youyu Li, Weiyi Ye, and Ou Zhang collected the data. Weiwei Quan, Youyu Li, Xuezhi Yang analyzed the data, and drafted the manuscript. Weiwei Quan, Youyu Li, and Xu Zhang contributed to rewriting and editing the final version of the manuscript.

### Peer Review

The peer review history for this article is available at https://publons.com/publon/10.1002/brb3.1798.

## Data Availability

The data that support the findings of this study are available from the corresponding author upon reasonable request.
